# Nutritional, Physicochemical, and Endogenous Enzyme Assessment of Raw Milk Preserved under Hyperbaric Storage at Variable Room Temperature

**DOI:** 10.1021/acsfoodscitech.2c00027

**Published:** 2022-05-31

**Authors:** Ricardo V. Duarte, Susana Casal, José A. Lopes da Silva, Ana Gomes, Ivonne Delgadillo, Jorge A. Saraiva

**Affiliations:** † LAQV-REQUIMTE, Departamento de Química, Universidade de Aveiro, 3810-193 Aveiro, Portugal; ‡ Universidade Católica Portuguesa, CBQF-Centro de Biotecnologia e Química Fina-Laboratório Associado, Escola Superior de Biotecnologia, Rua Diogo Botelho 1327, 4169-005 Porto, Portugal; § LAQV/REQUIMTE, Departamento de Ciências Químicas, Laboratório de Bromatologia e Hidrologia, Faculdade de Farmácia – Universidade do Porto, 4050-313 Porto, Portugal

**Keywords:** raw milk, hyperbaric storage, volatile compounds, free amino acids, viscosity, fatty acid profile

## Abstract

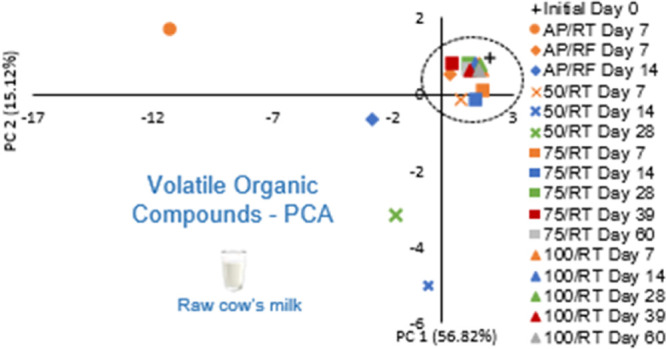

Raw milk (a highly perishable food) was preserved at variable room temperature (RT) under hyperbaric storage (HS) (50–100 MPa) for 60 days and compared with refrigeration (RF) under atmospheric pressure (AP) on quality, nutritional, and endogenous enzyme activity parameters. Overall, a comparable raw milk preservation outcome was observed between storage under AP/RF and 50/RT after 14 days, with similar variations in the parameters studied indicating milk degradation. Differently, even after 60 days (the maximum period studied) under 75–100/RT, a slower milk degradation was achieved, keeping most of the parameters similar to those of milk prior to storage, including pH, titratable acidity, total solid content, density, color, viscosity, and volatile organic and fatty acid profiles, but with higher free amino acid content, signs of an overall better preservation. These results indicate an improved preservation and enhanced shelf life of raw milk by HS/RT versus RF, showing HS potential for milk and highly perishable food preservation in general.

## 1 Introduction

Hyperbaric storage (HS) is a novel preservation methodology that employs milder pressure values (20–150 MPa) than the ones (400–600 MPa) commonly used in high-pressure processing (HPP). One of the reasons why HS is rising substantial research interest for food preservation is the fact that it results in significant energy reduction compared to refrigeration (RF) since it can be applied at variable room temperature (RT) with no energy spent in maintaining constant low temperatures as in RF, with energy being only required to pressurize and depressurize the vessel, where food would be stored.

In fact, Bermejo-Prada et al.^1^ estimated that compared to RF, storage of 800 kg of strawberry juice for 15 days at HS/RT would allow a 26-fold lower energy cost but would require so far higher investment in HS equipment. However, the equipment forecast in this study is based on the ones currently available for HPP, which are highly more complex and demanding, as they need to achieve fast and elevated pressures (up to 600 MPa), thereby requiring a more robust vessel that can endure higher pressures than the ones for HS.^1^ Also, Bermejo-Prada et al.^1^ reported an estimated reduction of almost 25.8-fold for HS in carbon footprint (per kg strawberry juice), resulting in a more sustainable preservation methodology that in return would account for negligible emission tax when compared to RF.

Another main reason for HS research interest is the potential considerable increment in food shelf life, with very interesting microbial results being reported for several types of food products. Initially, HS was studied for juice preservation as case a study, while recently other foods, including solid food matrices, are being evaluated. The HS studies on juices were carried out on strawberry juice (an acidic juice), having been found that most of the physicochemical parameters remained stable even under low pressures such as 25 MPa at 20 °C for 15 days, as the low pH acts synergistically with HS to restrain the microbial growth.^2^ Later, non-acid juices (melon and watermelon juice) at and above RT but for shorter storage periods, up to 60 h, have shown great stability in most of the parameters studied, requiring pressures of up to 50 MPa to achieve a similar preservation to RF, while pressures above 75 MPa allowed a greater microbial stability, even at 25–37 °C, causing microbial inactivation.^3^ Further, longer storage periods (10 days) were assessed in watermelon juice (50–100 MPa) and whey cheese (100 MPa) at RT, reporting an initial microbial growth inhibition under 50 MPa, while 75–100 MPa allowed microbial inactivation in both food products, resulting in an increased shelf life compared to RF.^4^ More recently, the feasibility to store fish/meat products under HS was studied for longer storage periods (up to 60 days), with promising results. Both food products revealed great microbial stability by HS above 50 MPa, with microbial reductions being verified in both endogenous and inoculated microorganisms.^5^ Thus, although HS at RT has proven its capability to control microbial growth, it is important to perform more insightful analysis in other important foods, such as milk, not only regarding the microbial quality but also the nutritional, physicochemical, and biochemical quality parameters, to gain further knowledge about the potential of HS to possibly substitute RF with prolonged shelf life.

Raw milk is a highly perishable food product with short shelf life due to its high nutritional profile, near-neutral pH, and high-water activity, resulting in a good environment for the development of several microorganisms, that jeopardize the overall quality and safety of milk as well as dairy products produced from it, thus requiring refrigerated storage to slow down the microbial growth, prior processing. So far, there are no results for the effect of HS for milk preservation, with the exception of a study carried out recently by our group,^6^ with raw milk stored under HS conditions, 75–100 MPa at RT showing considerable increased microbial stability at RT for 60 days, compared to RF, with microbial inactivation observed to undetectable counts for endogenous microbial load (up to more than 5 log units reduction) and inoculated surrogate and pathogenic microorganisms (*Escherichia coli*, *Listeria innocua*, and *Salmonella enterica*), as well as bacterial spores (*Bacillus subtilis* endospores) throughout storage. These results clearly indicate the great potential of HS for raw milk preservation, compared to RF, with potentially increased shelf life and microbial safety.

Therefore, in the present study, several raw milk quality/nutritional parameters and activity of endogenous enzymes were studied, with raw stored under HS (50–100 MPa) at variable RT (18–22 °C) and stored at atmospheric pressure (AP) at RF and RT for 60 days. An overall assessment in milk pH, titratable acidity, total solid content, density, color, viscosity, volatile organic and fatty acid profiles, lipid oxidation, total protein, soluble protein (SP), free amino acids (FAAs), and alkaline phosphatase and lactoperoxidase activities was performed and compared to the milk prior to storage and milk stored in the different storage conditions.

## 2 Materials and Methods

### 2.1 Sample Preparation and Storage Conditions

Cow raw milk was collected from a local dairy farm association company, kept under RF during transportation, and then packaged under aseptic conditions inside a laminar flow cabinet (BioSafety Cabinet Telstar Bio II Advance, Terrassa, Spain). The samples were double-packed in UV-light-sterilized, low-permeability polyamide–polyethylene commercial food packaging bags (90 μm, IdeiaPack, Comércio de Embalagens, LDA, Abraveses, Viseu, Portugal) and heat-sealed individually, avoiding as much as possible, leaving air inside.

Then, the packaged raw milk was stored under three different vessels at 50, 75, and 100 MPa at RT (18–22 °C) using an SFP FPG13900 Model (Stansted Fluid Power, Stansted, UK) system, equipped with pressure vessels of 30 mm inner diameter and 500 mm height, with a mixture of (40:60) propylene glycol and water used as the pressuring fluid. For comparison, the raw milk samples were also stored at RT and RF (4 °C) at AP (0.1 MPa) for 7, 14, 28, 39, and 60 days and kept in the dark to simulate the same conditions of HS samples. Once the raw milk stored under the different storage conditions was considered microbiologically unsuitable,^6^ the study of that storage condition was stopped.

### 2.2 Physicochemical Parameters

pH was measured directly in the sample at constant RT with a proper calibrated pH meter (Testo 205, Testo, Inc., New Jersey, USA). The total solid content and density were determined using a portable density and brix meter (Handheld Refractometer Atago, ATC-1E, Tokyo, Japan) at 20 and 15 °C, respectively. The titratable acidity of the raw milk samples was determined by titrating 5 mL of diluted raw milk (2 mL of milk in 3 mL of distilled water) to pH 8.4 with a previously standardized sodium hydroxide 0.01 M solution using an automatic titrator (TitroMatic 1S, Crison Instruments, S.A., Barcelona, Spain). The results were expressed as grams of lactic acid per liter of milk based on eq 1
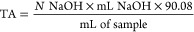
1

### 2.3 Color

The color parameters were measured using a Minolta Konica CM 2300d equipment (Konica MinoltaCM 2300d, Osaka, Japan), calibrated before each sample measurement. The color parameters were recorded in the CIELab system and directly computed through the original SpectraMagic NX software (Konica Minolta, Osaka, Japan) according to the International Commission on Illumination regulations: red/green color (a*), yellow/blue color (b*), and luminosity (L*) parameters. The color parameters L*, a*, and b* were measured, and the total color change (ΔE*) was calculated by eq 2
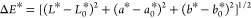
2where ΔE* represents the total color difference between a respective sample and the initial one prior to storage, with *L*_0_^*^, *a*_0_^*^, and *b*_0_^*^ representing the respective parameter at day 0.

### 2.4 Alkaline Phosphatase and Lactoperoxidase Activity

The alkaline phosphatase (ALP) activity was assayed with *p*-nitrophenylphosphate (*p*-NPP) as a substrate, as described by Negrão et al.,^7^ with some modifications (in this method, *p*-NPP is hydrolyzed in the presence of alkaline phosphatase to *p*-nitrophenol, resulting in an intense yellow color quantified at 405 nm). Initially, raw milk was mixed with 4 mM *p*-NPP in buffer solution (100 mM Tris-HCl, pH 9.5, 100 mM NaCl, and 5 mM MgCl_2_) and incubated for 30 min at 37 °C. The reaction was stopped by the addition of 2 M NaOH, and the *p*-nitrophenol released was measured at 405 nm using a microplate spectrophotometer (Multiskan GO Microplate Spectrophotometer, Thermo Scientific, Thermo Fisher Scientific, Waltham, Massachusetts, USA). The activity was expressed in ΔAbs_405nm/min_ (calculated as the slope of the curve relating Abs increment vs time).

The lactoperoxidase (LPO) activity assay was performed based on the method described by Marín et al.^8^ Briefly, raw milk was mixed with a solution of 0.325 mM ABTS (in 0.1 M sodium phosphate buffer, pH 6.0) and left for 30 min at 20 °C, and then 0.1 mM hydrogen peroxide was added and mixed quickly to initiate the reaction, with the absorbance (Abs_412nm_) measured for 1 min. The enzymatic activity was calculated as the slope of the curve relating Abs increment versus time and expressed as ΔAbs_412nm_ AU/min.

All enzymatic assays were performed in triplicates for each storage condition, with the residual activity calculated by eq 3
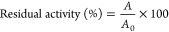
3where *A* is the enzymatic activity in raw milk samples after storage and *A*_0_ is the enzymatic activity of the sample at day 0.

### 2.5 Viscosity

Milk viscosity was assessed through a controlled stress rheometer (AR-1000, TA Instruments, New Castle, USA) equipped with a cone-and-plate geometry (acrylic cone, 6 cm diameter and 2° angle). Prior to the analysis, the samples were allowed to achieve a constant temperature (20 ± 0.5 °C) on the rheometer equipped with a fixed flat plate at the bottom for 300 s. A circulatory thermostatic bath (Circulating Bath 1156D, VWR International, Carnaxide, Portugal) was connected to this plate, ensuring that the target temperature was achieved and maintained. Before placing the samples on the rheometer plate, each sample was mixed gently and carefully transferred onto the rheometer measuring system, avoiding the trapping of air bubbles between the cone and the plate. Flow curves were obtained by applying a continuous stress ramp from 0 to 2 Pa for 3 min. Rheological results were monitored using a TA Instruments software package. The apparent viscosity measured at a shear rate of 300 s^–1^, within the Newtonian region, was used to compare among samples.

### 2.6 Volatile Organic Compounds

Volatile organic compound (VOC) profile determination was based on the method described by Yue et al.^9^ It was performed by headspace solid-phase microextraction, followed by gas chromatography–mass spectrometry (GC–MS). Raw milk (2 mL) was pipetted into 35 mL vials containing the internal standard (50 μL of cyclohexanone aqueous solution at 25 μg/mL) and immediately sealed with a metallic cap with silicon septum. After equilibration at 50 °C for 30 min, with agitation (500 rpm), the SPME fiber (DVB/CAR/PDMS; 50/30 μm; Supelco Inc.) was exposed to the sample’s headspace for 30 min at 50 °C for adsorption of volatile compounds. The fiber was inserted in the injection port of the GC equipment, an Agilent GC-7890 gas chromatographer, equipped with a an Agilent 5977B mass spectrometer, and a DB-5 MS Capillary GC column (30 m × 0.25 mm I.D. × 0.25 μm film thickness, Agilent, USA). The injector port was heated to 260 °C, and injections were performed in the splitless mode with helium at a linear velocity of 1 mL/min. The oven temperature was set at 35 °C for 5 min, increasing to 100 °C at a rate of 4 °C/min, followed by an increase of 10 °C/min until 225 °C, and held for 0.25 min (total of 33.5 min). The ion source and interface temperatures were maintained at 230 and 280 °C, respectively, and the electron impact ionization mass spectra were recorded with an ionization energy of 70 eV. Mass spectra were scanned from 20 to 350 *m*/*z* in the full scan mode. Identification of the volatile compounds was based on computer-matching with the reference mass spectra of the MS library of the National Institute of Standards and Technology 2011 (NIST 11), retention times, retention index, and with individual standards when available. Using cyclohexanone as the internal standard equivalent basis, the volatile profile was semi-quantitatively determined from the full scan areas, and the results were expressed in μg of the internal standard equivalents per mL of milk.

### 2.7 Fatty Acid Profile

For the determination of fatty acid profile, a method similar to that described by Sobral et al.^10^ was performed. Briefly, 100 μL of the internal standard solution (10 mg/mL of undecanoin, C11:0 triglyceride, in heptane) was evaporated to dryness under a gentle stream of nitrogen (Stuart, Staffordshire, USA), and 1 mL of milk was added, followed by the addition of isopropanol (2 mL) for protein precipitation, cyclohexane (2 mL), and NaCl aqueous solution (1%) (1.5 mL). After agitation and centrifugation (5000 rpm, 5 min), the supernatant was collected, evaporated under a nitrogen stream at RT, and redissolved with heptane (2 mL). For the preparation of fatty acid methyl esters (FAME), 2 M potassium hydroxide (200 μL) was added, and the samples were carefully vortexed for 1 min. Finally, 50 μL of injection standard [20 mg/mL of methyl tridecanoate (C13:0 methyl ester)] was added. The hexane layer containing FAME was transferred into 1 mL GC vials.

The FAME profile was analyzed using a gas chromatograph (Chrompack CP-9001 model, the Netherlands) with flame ionization detection (FID). 1 μL of the extracted FAME was injected into the GC system, with the injector and detector temperatures set to 250 and 270 °C, respectively. Separation of the fatty acids was achieved on a Select FAME (50 m × 0.25 mm × 0.25 μm) column (Agilent, USA) using helium as the carrier gas (pressure of 140 kPa), heated from 120 °C (3 min hold) to 220 °C (5 min hold) at a rate of 3 °C/min. Fatty acid identification and FID calibration were accomplished with a certified reference standard mixture (TraceCERT—Supelco 37 component FAME mix, USA), and the results were expressed in relative percentages of their FAMEs.

### 2.8 Secondary Lipid Oxidation Byproducts

Lipid oxidation was determined by malondialdehyde (MDA) quantification using the 2-thiobarbituric acid reactive substances (TBARS) method with adaptations.^11^ Briefly, 1 mL of raw milk was mixed with 2 mL of 7.5% trichloroacetic acid and then vortexed for approximately 60 s, followed by centrifugation at 4000*g* at 4 °C for 20 min (Universal 320-R, Hettich Group, Tuttlingen, Germany). After filtration (Whatman no. 1), 1 mL of the resulting extract was added to 1 mL of 46 mM 2-thiobarbituric acid, vortexed and immersed in boiling water for 40 min, and then cooled down in cold water. Triplicates were measured using a microplate spectrophotometer (Multiskan GO Microplate Spectrophotometer, Thermo Scientific, Thermo Fisher Scientific, Waltham, Massachusetts, USA) with a Brand plate of 96 wells, at 532 nm. Standard solutions of MDA in 7.5% trichloroacetic acid were prepared from 1,1,3,3-tetramethoxypropane, and a calibration curve was prepared at a concentration ranging from 0.2 to 10 μg/L. TBARS results were expressed as μg of MDA per mL of milk.

### 2.9 Protein Profile

The overall protein profile was assessed by determining the total nitrogen (TN) using the Kjeldahl method, SP by the Bradford method, and FAAs using the EZ:Faast Amino Acid Analysis Kit available for GC-FID. The micro-Kjeldahl procedure was performed with a Kjeltec system 1002 Distilling unit (Tecator, Sweden), and the crude protein content was determined by multiplying the total nitrogen content by 6.38 (AOAC Official Method 2001.14, 2002). The total SP was determined based on the Bradford method^12^ with few modifications. Initially, milk was diluted in distilled water (1:100 v/v), followed by centrifugation at 4000*g* at 4 °C for 15 min (Universal 320-R, Hettich Group, Tuttlingen, Germany). Then, 50 μL of the supernatant was added to 250 μL of dye Coomassie Blue G25 in a microplate, shaken for 30 s, and incubated for 20 min at RT. The absorbance was measured at 595 nm (Microplate Spectrophotometer Multiskan GO, Thermo Scientific, Waltham, MA, USA), and SP was expressed in mg per 100 mL of milk. A calibration curve was prepared using BSA as the standard at concentrations ranging from 0 to 0.5 mg/mL. For FAA determination and quantification, the milk was centrifuged (17,000*g* at 4 °C for 5 min), and the supernatant was collected and centrifuged again. The secondary supernatant (100 μL) was used for the analysis of FAAs using the EZ:Faast Amino Acid Analysis Kit (GC-FID),^13^ and the results were expressed in nmol per mL of milk.

### 2.10 Statistical Analyses

All experiments and analyses were carried out in triplicate. Analysis of variance was performed under all the different storage conditions, followed by a multiple comparison post hoc test, Tukey’s HSD test, at a 5% level of significance. Additionally, principal component analysis (PCA) was performed in order to identify the statistical patterns in VOC data.

## 3 Results and Discussion

### 3.1 Physicochemical Parameters

The pH, titratable acidity, total solid content, and density were assessed in all samples under the different storage conditions (Table 1). The initial raw milk presented a pH value of 6.68 ± 0.01, which is within the values reported in the literature.^14^ As expected, storage conditions that allowed fast and considerable microbial growth^6^ resulted in an increasing acidity (*p* < 0.05), just after 7 days, in the case of AP/RT with a pH of 4.14 ± 0.02 (Table 1). A less pronounced but significant (*p* < 0.05) decrease to 6.53 ± 0.01 for storage under AP/RF after 14 days and to 6.43 ± 0.01 at 50/RT after 28 days was also observed. The raw milk stored under 75 and 100 MPa also presented a slight decrease in pH throughout storage; despite being statistically significant (*p* < 0.05), the decrements were much smaller, with the pH decreasing slightly to around 6.51 ± 0.2 for both storage conditions after 60 days (compared to the initial value, 6.68).

**Table 1:** pH, Titratable Acidity (g Lactic Acid/L), Total Solids (%), Density (g/mL), Color, Viscosity (mPa·s), Lipid Oxidation (μg MDA/mL), and Residual Activity of Alkaline Phosphatase and Lactoperoxidase (%) Parameters of Raw Milk Prior to Storage (Initial) and Stored under the Different Conditions (AP/RT, AP/RF, and 50, 75, and 100/RT)^a^

condition	Initial	AP/RT	AP/RF		50 MPa/RT			75 MPa/RT					100 MPa/RT				
days	0	7	7	14	7	14	28	7	14	28	39	60	7	14	28	39	60
pH	6.68j	4.14a	6.64hij	6.53c	6.66ij	6.59efgh	6.43b	6.63ghi	6.60efgh	6.57ef	6.56ef	6.51cd	6.61fghi	6.58efg	6.57ef	6.55de	6.51cd
titratable acidity (g lactic acid/L)	1.73a	11.66f	1.77ab	2.22de	1.79ab	2.01bcd	2.41e	1.87abc	2.08cd	2.09cd	2.13d	2.24de	1.76ab	2.02bcd	2.13d	2.22de	2.26de
total solids (%)	11.83d	9.17a	10.42b	10.67bc	11.83d	11.67cd	11.58cd	11.17bcd	12.17d	11.97d	11.33bcd	11.25c	11.25bcd	11.92d	12.00d	11.48bcd	11.58cd
density (g/mL)	1.037b	1.029a	1.036b	1.033ab	1.038b	1.038b	1.038b	1.036b	1.038b	1.038b	1.033ab	1.033ab	1.036b	1.037b	1.038b	1.034ab	1.036b

Color																	
L*	54.11ab	55.79b	54.21ab	53.40a	53.81ab	53.59ab	52.77a	53.54ab	53.12a	53.73ab	53.09a	52.81a	53.39a	53.96ab	54.14ab	52.92a	52.68a
a*	–0.77bc	–0.89ab	–0.78abc	–0.82abc	–0.86abc	–0.83abc	–0.87ab	–0.82abc	–0.83abc	–0.89a	–0.86abc	–0.89a	–0.75c	–0.79abc	–0.85abc	–0.82abc	–0.84abc
b*	2.74a	2.75a	2.78a	2.65a	2.52a	2.51a	2.52a	2.62a	2.62a	2.81a	2.59a	2.78a	2.96a	2.93a	2.79a	2.86a	3.11a
Δ*Ε*	NP	1.70ef	0.56a	0.71abc	0.41a	0.59ab	1.37cdef	1.07bcde	1.00abcd	0.73ab	1.03abcde	1.89f	0.79abc	0.48a	0.77abc	1.19bcde	1.48def
viscosity (mPa·s)	2.87a	NP	31.12c	NP	4.99ab	10.63d	NP	2.84a	2.80a	2.76a	2.74a	3.00a	2.96a	2.91a	2.83a	2.80a	2.96a
lipid oxidation (μg MDA/mL)	0.83ab	1.18c	0.72a	0.78ab	0.89abc	0.93ab	0.91ab	0.82ab	0.74a	0.80ab	0.88ab	1.03bc	0.75a	0.73a	0.78ab	0.76a	0.84ab
alkaline phosphatase (%)	100e	36.2a	81.0bcde	85.5de	68.1bcd	59.6ab	63.8bcd	74.6bcd	84.9cde	72.7bcd	75.9bcde	57.5ab	73.0bcd	65.9bcd	66.1bcd	63.6bcd	61.5bc
lactoperoxidase (%)	100fg	23.8b	79.0e	47.0bc	32.9b	4.6a	NP	112.7gh	82.6ef	84.3ef	73.5de	55.7cd	122.5h	119.2h	87.4ef	82.7ef	80.9e

a Different letters (a–j) indicate significant differences (*p* < 0.05) between the different conditions for each parameter. Standard deviation is at least below 10% of the mean value and thus is not displayed in the table. (NP—parameters not performed under these conditions).

Regarding titratable acidity, the initial value observed was similar to the values reported in the literature, 1.73 ± 0.02 g/L.^15^ A substantial increase was observed for samples stored under AP/RT, with titratable acidity increasing to 11.66 ± 0.18 g/L after 7 days (Table 1). As mentioned, due to the highly perishable nature of milk, high water activity, and neutral pH, it provides a good environment for microbial growth, which results in increasing organic acid concentrations that are responsible for higher acidity,^15^ as observed. Initially on day 7, the acidity of all the other storage conditions increased slightly (*p* > 0.05) compared to samples prior to storage. The milk acidity continued to increase for all storage conditions, however at different rates, with samples stored under AP/RF and 50/RT reaching similar values, just after 14 and 28 days, 2.22 ± 0.16 and 2.41 ± 0.05 g/L, respectively, while when stored under 75 and 100/RT, the milk reached a maximum of 2.24 ± 0.13 and 2.26 ± 0.03 g/L after 60 days, respectively.

For total solids, the samples prior storage had a value of 11.83 ± 0.63%, similar to the values reported in the literature, 9.7–12.5%.^16^ Under AP/RT and AP/RF after 7 and 14 days, respectively, a significant decrease (*p* < 0.05) was observed, reaching a minimum of 9.17 ± 0.26% and 10.67 ± 0.38%, respectively. Storage under pressure maintained a similar TS value throughout the storage period with slight variations observed (*p* > 0.05), with final values of 11.25 ± 0.42 and 11.58 ± 0.14 after 60 days at 75 and 100/RT, respectively.

As for density, the values obtained were within the ones observed in the literature for refrigerated raw milk, which should be between 1.023 and 1.040 g/mL, as values outside this range may indicate adulteration, such as water addition.^15^ The only variation (*p* < 0.05) observed within all the storage conditions was for AP/RT that presented a significant decrease from 1.036 ± 0.003 to 1.029 ± 0.001 g/mL after 7 days, with storage under RF and HS resulting in no modifications in milk density (*p* > 0.05).

### 3.2 Color

The color parameters L*, a*, and b* were monitored in milk stored under the different conditions, and the total color change (ΔE*) was calculated, followed by comparison to the initial values of milk prior to storage (Table 1). Raw milk presented L*, a*, and b* values of 54.11 ± 0.81, −0.77 ± 0.04, and 2.74 ± 0.24, respectively. L* (lightness values) ranged from 55.79 ± 0.03 (AP/RT at day 7) to 52.68 ± 0.14 (100/RT at day 60), with no significant changes (*p* > 0.05) observed compared to the initial value.

Overall, HS presented a slight decreasing tendency (*p* > 0.05) in the raw milk L* parameter, but with no statistical significance (*p* > 0.05). Regarding a* (greenness), the only significant variation was observed under 75/RT at days 28 and 60 (*p* < 0.05), to values of −0.89 ± 0.02, for both periods. As for b* (yellowness), no changes were observed under the different storage conditions (*p* > 0.05), ranging from 2.51 ± 0.04 to 3.09 ± 0.04. In milk HPP studies, the L* parameter is usually the most affected (*p* < 0.05), decreasing after processing,^17^ which could be due to disintegration of casein micelles into smaller fragments that increase the translucency of milk, thus affecting this color parameter. However, the HPP studies apply significant higher pressures compared to HS, which in the present work presented only a slight decrease (*p* > 0.05) throughout the storage.

Relatively to the overall color changes, ΔE*, at the 7th day of storage, only samples stored at AP/RT showed a significant increase (*p* < 0.05) to values of 1.70 ± 0.03, compared to storage under AP/RF, 0.56 ± 0.16. On the 14th day, a slight increase (*p* > 0.05) was observed for most storage conditions, compared to the respective storage at day 7, which tended to increase as the time passed. On the 28th day, samples at 50/RT presented a significantly increased ΔE* value of 1.37 ± 0.30, while storage at 75 and 100/RT maintained a ΔE* value similar to that of day 7. However, at the end of the storage, at day 60, the values increased to 1.89 ± 0.17 and 1.48 ± 0.15 under 75 and 100/RT, respectively, possibly related to the observed decrease in the L* parameter in these samples. According to Drlange,^18^ all of the ΔE* values for samples in this study are considered to have a “small difference” (0.5 < ΔE* < 1.5) perceptible by the consumer’s eyes compared to the initial raw milk color, with samples under AP/RT at day 7 and 75/RT at day 60 having “distinct differences” (1.5 < ΔE* < 3).

### 3.3 Alkaline Phosphatase and Lactoperoxidase Activity

Alkaline phosphatase (ALP) is an enzyme naturally present in milk, mainly bounded to the fat globule membrane, that can catalyze the hydrolysis of phosphate monoesters, yielding phosphate and the corresponding alcohol. ALP is also commonly used as a standard assay for rapid validation of the milk pasteurization process as it is slightly more resistant to thermal treatment than the non-sporogenic pathogenic microorganisms present in milk.^19^ The residual activity of ALP under all storage conditions throughout storage is described in Table 1. After 7 days, the ALP activity was reduced for all the storage conditions, reaching a significant decrease (*p* < 0.05) of 35%, at AP/RT with the rest of the storage conditions presenting residual activities similar to AP/RF (around 81%). Overall, after 7 days of storage, the ALP activity tended to decrease slightly over time, but globally without statistical significance (*p* > 0.05), compared to the value at day 7. Similarly, Fidalgo et al.^20^ also observed a decrease in acid phosphatase in Atlantic salmon under 75 MPa at 25 °C after 25 days to 23% opposingly to storage under 60 MPa at 10 °C after 30 days, which maintained the acid phosphatase activity stable (around 111%).

LPO is also an enzyme commonly present in milk, being one of its indigenous antimicrobial agents. This enzyme catalyzes the oxidation reactions in the presence of hydrogen peroxide and helps the production of products with a wide antimicrobial activity, such as pseudohalogens, thiocyanates, or halogens.^21^ At AP/RT, the LPO activity was significantly reduced (*p* < 0.05) to 24% of its initial value after 7 days (Table 1). Under AP/RF, the LPO activity decrease was slower, but still significant (*p* < 0.05) when compared to its initial activity to around 79 and to 47% after 7 and 14 days, respectively. Storage under 50/RT presented a behavior alike AP/RT, with a more pronounced reduction (*p* < 0.05) in the LPO activity up to 33 and 5% of the initial value after 7 and 14 days, respectively. This enzyme might be more susceptible to the changes observed under these storage conditions, AP/RT, AP/RF, and 50/RT, namely, high microbial activity, decrease in pH, and increase in acidity, which all together can promote LPO denaturation and/or reduce its activity.^22^

Storage under 75 and 100/RT presented overall a much better maintenance in LPO residual activity throughout storage, compared to the other storage conditions performed. At day 7, both storage conditions presented an increased LPO activity, compared to the initial one, to values around 113% (*p* < 0.05) and 123% (*p* < 0.05) for 75 and 100 MPa, respectively. After 14 and 28 days, despite the decrease in LPO activity observed for both storage conditions, no significant variations were observed compared to the initial LPO activity (*p* < 0.05). Overtime, the LPO activity was slightly more affected during storage under 75/RT, ending at day 60 with an activity of 56% of the initial value, while storage under 100/RT maintained an LPO residual activity similar to the one observed at days 28 and 39, around 80% of the initial value, at the end of the storage period (*p* < 0.05).

Knowledge about the HPP effect, especially for low and mild pressures, in enzyme activity is scarce, and the HPP effect is not always straightforward, being dependent in several variables, such as HPP pressure level, duration, temperature, pH, and the matrix environment, and can be specific for a determined enzyme.^23^ In HPP studies, LPO is described as highly resistant without significant inactivation even after 60 min at 400 MPa or 15 min at 700 MPa.^24^ Despite that, the prolonged HS effect in the enzymatic activity is scarcely discussed in the literature, with only two works having evaluated peroxidase (POD) activity during HS/RT in watermelon and strawberry juice, for 10 and 15 days, respectively.^4a,25^ Bermejo-Prada and Otero^25^ reported a constant residual POD activity throughout storage, decreasing only to 85% under 200 MPa at 20 °C. As in Pinto et al.’s^4a^ work, a significant reduction overtime in POD residual activity was reported, to values of 54.6 and 16.8% after 10 days under 75 and 100 MPa, respectively. In the present study, milk stored at 100/RT resulted in the decrease of LPO residual activity to 80% on the 28th day of storage, remaining stable until the end of the storage period, possibly retaining the LPO antimicrobial activity.

### 3.4 Viscosity

Apparent viscosity was determined for all studied storage conditions (Table 1), with the exception for samples stored for 7 days at AP/RT and for 14 days at AP/RF since these samples presented visible signs of spoilage (clots, swelling, and increased viscosity). The initial viscosity value for raw milk was similar to values reported in the literature, 2.87 ± 0.20 mPa·s.^26^ Storage at AP/RF presented an almost 10-fold increase (*p* < 0.05) in milk viscosity to 31.12 ± 5.98 mPa·s after 7 days, while under HS conditions (50, 75, and 100/RT), it remained unchanged (*p* > 0.05) for this storage period. After 14 days, samples under 50/RT presented a viscosity of 10.63 ± 1.39 mPa·s (*p* < 0.05), while samples under 75 and 100/RT showed no changes in viscosity (*p* > 0.05) throughout the entire storage period, with values of 3.00 ± 0.02 and 2.96 ± 0.07 mPa·s, respectively, at the end of the storage (60 days). The considerable microbial growth observed in samples at AP/RF and 50/RT^6^ may induce changes in milk composition, such as a decrease in pH and an increase in extracellular proteases and polymeric substances released by lactic acid bacteria, that have shown to increase the viscosity of milk.^27^ Several studies have shown that HPP causes an increase in milk viscosity, directly dependent on treatment intensity from pressures above 200 MPa for 30 min, with a slight increase in milk viscosity also observed in treatments below that pressure.^28^ These changes are mostly related to the HPP effect in casein micelles, promoting changes in caseins shape, from spherical to non-spherical, micelle disruption, or even reduction in particle size.^28^ Apparently, under HS at 75 and 100 MPa, such changes seem to not to occur, or at least not at a level enough to cause observable changes in viscosity.

### 3.5 Volatile Organic Compounds

A total of 19 VOCs were identified in almost all samples, mostly free fatty acids (FFAs) and their ethyl esters, alcohols, and aldehydes (Table 2). FFAs were the most abundant VOCs in the initial raw milk (*n* = 5), namely, acetic, butanoic, hexanoic, octanoic, and decanoic acids, followed by 3-hydroxybutan-2-one, similar to what is reported for milk in the literature.^9^ In lower concentrations, some ethyl esters (*n* = 3), alcohols (*n* = 2), and aldehydes (*n* = 2) were also found, with 3-methylbutanal being detected only in the initial raw milk, prior to storage.

**Table 2:** VOCs of Raw Milk Prior to Storage (Initial) and Stored under the Different Conditions (AP/RT, AP/RF, and 50, 75, and 100/RT) Expressed in μg of the Internal Standard Equivalents/mL^a^

storage condition	Initial	AP/RT	AP/RF		50 MPa/RT		
days	0	7	7	14	7	14	28
**FFAs**	0.63ab	6.42g	1.76d	5.05f	1.74cd	3.05e	3.56e
acetic acid	0.08a	2.80c	0.74a	2.21bc	0.55a	1.51b	1.99b
butanoic acid	0.18ab	0.96f	0.41bcd	0.82ef	0.45cd	0.61de	0.50d
hexanoic acid	0.26ab	2.06	0.41abc	1.53e	0.51bcd	0.68cd	0.81d
octanoic acid	0.10ab	0.63d	0.14ab	0.37c	0.15ab	0.18abc	0.17ab
nonanoic acid	nd	0.04a	0.04a	0.04a	0.03a	0.03a	nd
decanoic acid	0.02a	0.15e	0.04abc	0.14de	0.04abc	0.04abc	0.10cde
**esters**	0.21a	6.87d	0.64ab	1.11bc	0.47ab	0.45ab	1.64c
ethyl acetate	0.14abcd	1.43f	0.29cde	0.32de	0.14abc	0.21abcd	0.45e
ethyl butanoate	0.05a	1.80c	0.29ab	0.49ab	0.30ab	0.17ab	0.66b
ethyl hexanoate	0.02a	3.49b	0.06a	0.19a	0.04a	0.07a	0.39a
ethyl octanoate	nd	0.70b	nd	0.10a	nd	nd	0.12a
ethyl decanoate	nd	0.18b	nd	0.02a	nd	nd	0.02a
**alcohols**	0.08a	2.91g	0.57cdef	0.65def	0.32abcdef	0.68ef	0.71f
3-methylbutan-1-ol	0.04a	2.68d	0.57bc	0.61c	0.25ab	0.23a	0.35abc
2-methyl-1-butanol	nd	0.31b	nd	nd	nd	0.08a	nd
butane-2,3-diol	0.04a	nd	nd	0.04a	0.07a	0.37b	0.37b
2-ethylhexan-1-ol	nd	nd	nd	nd	nd	nd	nd
**aldehydes**	0.04ab	0.06ab	nd	0.03ab	0.06ab	0.24c	0.14bc
3-methylbutanal	0.03	nd	nd	nd	nd	nd	nd
hexanal	0.01a	0.06ab	nd	0.03a	0.06ab	0.24c	0.14bc
**ketones**							
3-hydroxybutan-2-one	0.48cd	nd	0.12ab	0.09a	0.55d	0.06a	0.04a
**others**							
toluene	nd	nd	nd	nd	nd	0.26b	nd

storage condition	75 MPa/RT					100 MPa/RT				
days	7	14	28	39	60	7	14	28	39	60
**FFAs**	0.60ab	0.78ab	0.99abc	1.37bcd	1.05acbcd	0.64ab	0.62ab	0.55a	0.81ab	0.72ab
acetic acid	0.15a	0.17a	0.21a	0.48a	0.22a	0.15a	0.10a	0.05a	nd	nd
butanoic acid	0.13a	0.20ab	0.24abc	0.24abc	0.23abc	0.20ab	0.17a	0.19ab	0.26abc	0.23abc
hexanoic acid	0.10a	0.24ab	0.35ab	0.32ab	0.29ab	0.18a	0.19ab	0.18a	0.27ab	0.31ab
octanoic acid	0.04a	0.10ab	0.14ab	0.27bc	0.26bc	0.08ab	0.12ab	0.10ab	0.20abc	0.14ab
nonanoic acid	0.14b	0.03a	0.02a	0.02a	nd	nd	nd	nd	nd	nd
decanoic acid	0.04abc	0.05ab	0.04ab	0.08abcd	0.05abc	0.02ab	0.03ab	0.02ab	0.08bc	0.05abc
**esters**	0.20a	0.20a	0.14a	0.37a	0.15a	0.19a	0.17a	0.11a	0.07a	0.07a
ethyl acetate	0.14abc	0.17abcd	0.05a	0.26bcd	0.07ab	0.19abcd	0.17abcd	0.11abc	0.07a	0.07a
ethyl butanoate	0.06a	0.03a	0.07a	0.06a	0.05a	nd	nd	nd	nd	nd
ethyl hexanoate	nd	nd	0.02a	0.04a	0.06a	nd	nd	nd	nd	nd
ethyl octanoate	nd	nd	nd	nd	nd	nd	nd	nd	nd	nd
ethyl decanoate	nd	nd	nd	nd	nd	nd	nd	nd	nd	nd
**alcohols**	0.11ab	0.31abcdef	0.54bcde	0.49abcdef	0.35abcdef	0.17abcd	0.23abcd	0.17abc	0.27abcde	0.13ab
3-methylbutan-1-ol	0.11a	0.21a	0.35abc	0.32abc	0.17a	0.13a	0.14a	0.10a	0.13a	0.07a
2-methyl-1-butanol	nd	0.10a	0.13a	0.13a	0.07a	0.06a	0.09a	0.06a	0.06a	0.04a
butane-2,3-diol	nd	nd	0.02a	0.03a	0.05a	nd	nd	nd	nd	nd
2-ethylhexan-1-ol	nd	nd	nd	nd	0.08a	nd	nd	nd	0.08a	0.03a
**aldehydes**	0.02a	0.03ab	0.03a	0.04ab	0.05ab	0.02a	0.03a	0.03a	0.05ab	0.04ab
3-methylbutanal	nd	nd	nd	nd	nd	nd	nd	nd	nd	nd
hexanal	0.02a	0.03a	0.03a	0.04ab	0.05ab	0.02a	0.03a	0.03a	0.05ab	0.04ab
**ketones**										
3-hydroxybutan-2-one	0.28abc	0.43cd	0.32bc	0.61d	0.23abc	0.08ab	0.06a	0.12ab	0.09ab	0.15ab
**others**										
toluene	nd	0.22b	0.02a	0.04a	0.03a	0.02a	0.02a	0.02a	0.04a	0.03a

a Different letters (a–f) indicate significant differences (*p* < 0.05) between the different conditions. In general, the standard deviation is at least below 10% of the mean value and thus is not displayed in the table. (nd—not detected).

Storage under AP/RT at day 7 resulted in significant changes (*p* < 0.05) in the major VOC classes, except for aldehydes, despite the slight increase in hexanal concentration. Overall, a significant increment (*p* < 0.05) in all FFAs was observed, almost up to 10-fold for acetic, butanoic, hexanoic, and decanoic acids, which can result mainly from the microbial action and lipase activity on fatty acids, and in a smaller degree, degradation of lactose and amino acids, that all together can be responsible for perceptible rancid flavor in milk.^14^ Esters were also more abundant (*p* < 0.05) in samples stored under AP/RT at day 7, when compared to the initial milk, which was particularly more pronounced for ethyl acetate, butanoate, and hexanoate. Additionally, ethyl octanoate and decanoate that were absent in the initial milk were now detected abundantly. The content in alcohols also increased considerably (*p* < 0.05) compared with milk prior to storage, especially for 3-methylbutan-1-ol (64-fold higher), with 2-methyl-1-butanol being now present. Both alcohols and esters can influence the flavor of dairy products when present in high concentrations, with alcohols being mainly derived from amino acid metabolism or fermentation of lactose and esters from esterification of short-chain alcohols and FFAs, both potentially indicating high microbial and enzymatic activities,^29^ which is coherent to what was reported for this storage condition (microbial levels above the acceptable level (≥5.5 log CFU/mL) for AP/RT samples at day 7).^6^ In what concerns aldehydes, for these samples only hexanal was found, showing a significant 5-fold increase under AP/RT (*p* < 0.05) that may derive from unsaturated fatty acids oxidation.^30^

At the 7th day of storage under AP/RF, the evolution of milk VOC profile was similar to AP/RT samples; however, the increments of the main VOC occurred at a slower rate, namely, for most FFA and esters, as under low temperature, the microbial growth and enzymatic activity are slowed down, as it was observed in a previous study regarding the microbial evolution between these two storage conditions, AP/RF and RT (around 5 log units for AP/RF after 7 days).^6^ After 14 days, the differences were more pronounced, with all FFAs and 3-methylbutan-1-ol presenting significantly higher concentrations (*p* < 0.05), when compared to milk prior to storage, with ethyl octanoate and decanoate now present (microbial counts reaching values above the acceptable limit).^6^

Under HS at the lowest pressure (50 MPa), the VOC concentration was comparable (*p* > 0.05) with AP/RF samples for the same storage period, with the exception for total FFAs, that were statistically found at a lower concentration (*p* < 0.05), while aldehydes were considerably higher (*p* < 0.05) when compared to the corresponding AP/RF samples. Although these samples presented a slower degradation rate pattern overall when compared to AP/RF, they presented significant increases (*p* < 0.05) in all identified VOCs when compared to the initial milk, with the presence of ethyl octanoate and decanoate being detected at the 28th day of storage, possibly resulting from the increased microbial load observed for this storage condition at the end of the storage period (above the acceptable limit).^6^ Toluene is a common compound reported in milk and dairy products, resulting from β-carotene degradation, detected in HS samples.

As for the upper pressures (75 and 100 MPa), a better overall preservation of the raw milk VOC profile was achieved, even after 60 days, compared to the initial one, for all FFAs, esters, alcohols, and aldehydes, with the exception for 3-hydroxybutan-2-one, whose concentration decreased considerably, especially (*p* < 0.05) under 50 and 100/RT. Between storage under 75 and 100/RT, the latter one presented a VOC profile more similar to that of the initial milk, with lower changes in all FFAs, with no nonanoic acid formation being detected (similar to milk prior storage) and also relatively for esters (only ethyl acetate was present, in low concentration), without the formation of fatty acids and ethyl esters, thus possibly indicating a better preservation of raw milk under these conditions. The overall alcohol content remained low and constant under 100/RT (*p* < 0.05), despite the formation in low concentrations of 2-methyl-1-butanol and 2-ethylhexan-1-ol. As far as the authors are aware, the information available regarding the effect of low pressures for extended periods on VOC of foods is very scarce and absent at variable RT. Anyway, for the sake of comparison, the results observed in the present work are in accordance with that reported by Fidalgo et al.,^31^ which observed a similar fresh salmon-like VOC profile for samples stored under 60 MPa at 10 °C up to 30 days. Regarding raw milk under variable RT, a slower matrix degradation evolution for both 75 and 100/RT for 60 days was observed compared to the sample prior to storage and a much better preservation of the VOC profile than those under AP/RF, which may also indicate a better control in microbial and enzymatic parameters.

**Figure 1:**
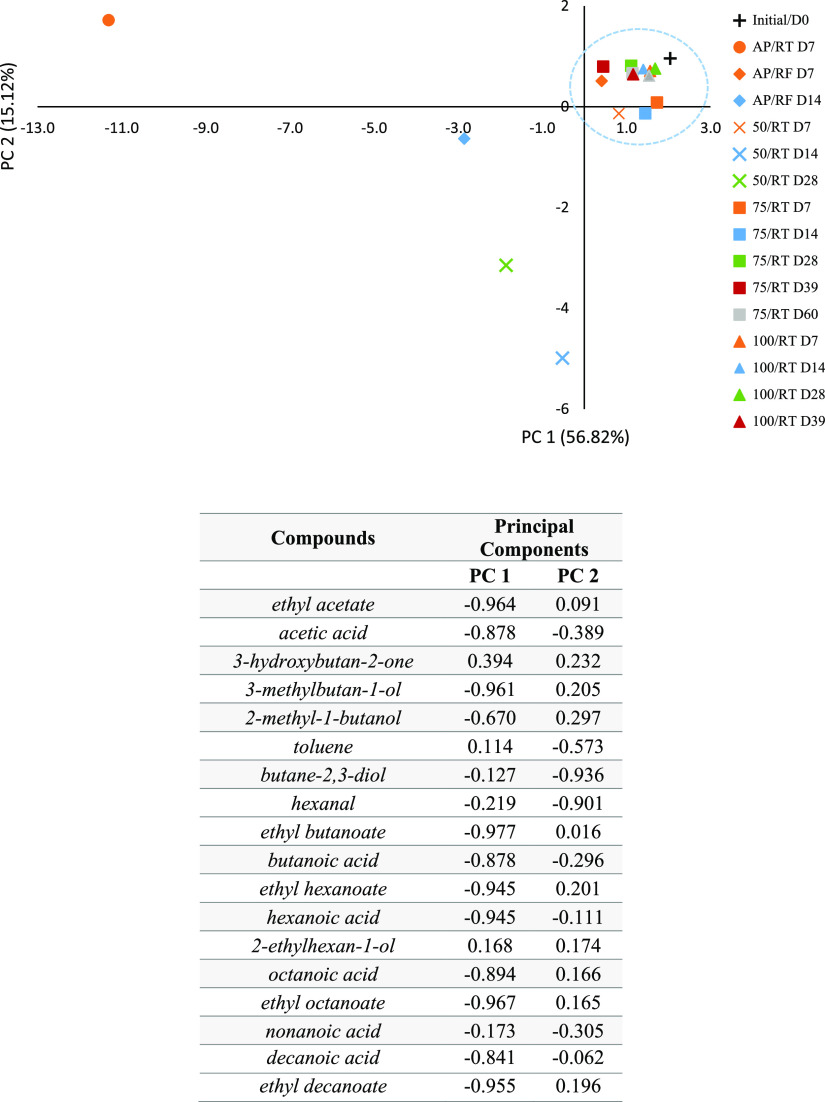
PCA score plot of the VOCs of raw milk prior to storage (initial) and stored under the different conditions (AP/RT, AP/RF, and 50, 75, and 100/RT). The same storage periods have the same color, while the same storage conditions have the same symbol. Below are also the loadings of the variables in the first two PCAs.

**Figure 2:**
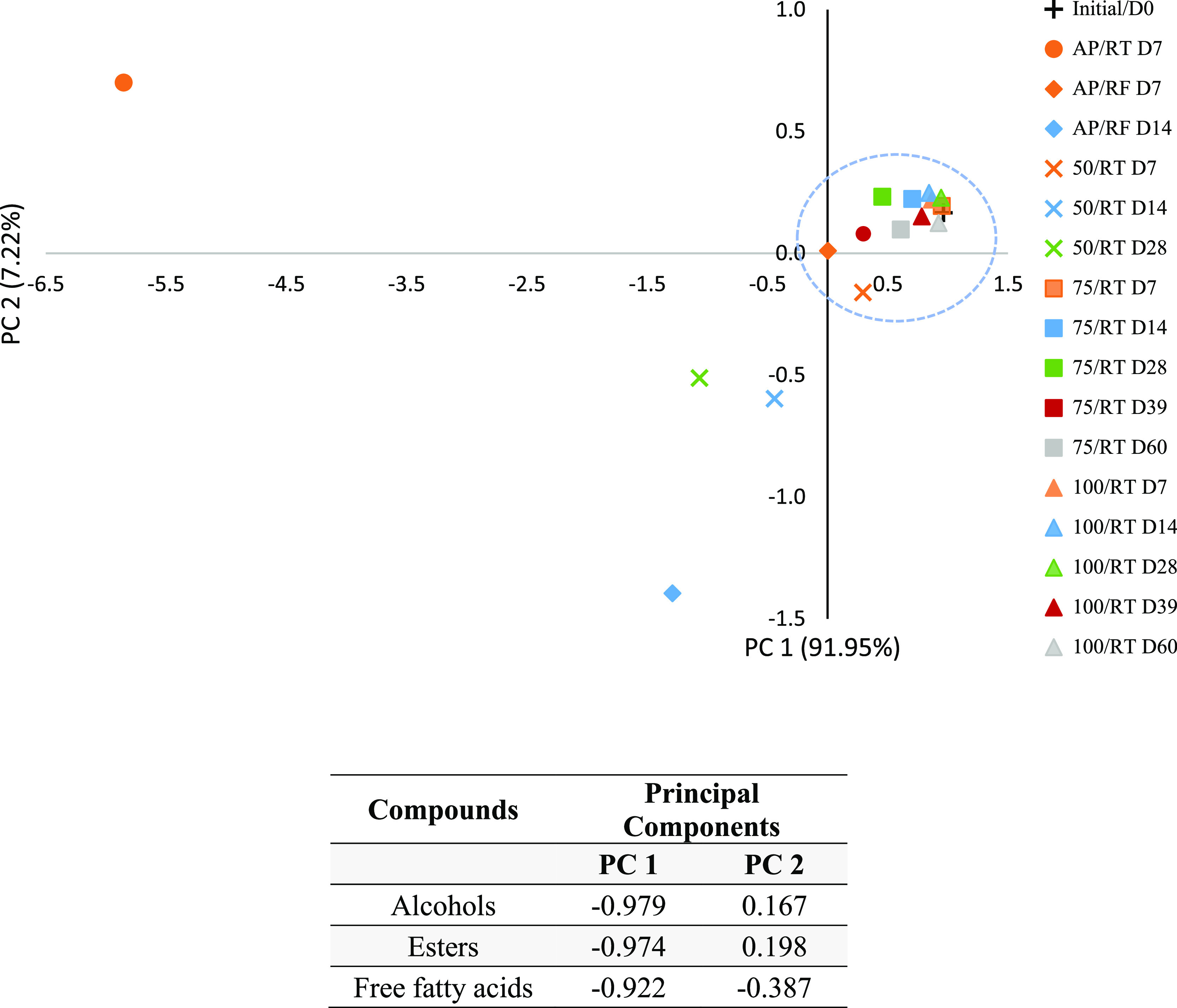
PCA score plot of the VOC major classes (FAA, esters, and alcohols) of raw milk prior to storage (initial) and stored under the different conditions (AP/RT, AP/RF, and 50, 75, and 100/RT). The same storage periods have the same color, while the same storage conditions have the same symbol. Below are also the loadings of the variables in the first two PCAs.

The complete set of VOC data from samples stored under the different storage conditions was subjected to multivariate statistical analyses, and the results from PCA are shown in Figure 1, which presents the scores and loadings, which explain 71.94% of the total variance with 56.82% of the total variance for PC 1 and 15.12% for PC 2. Compounds that scored positive on PC 1 are more associated with the initial milk prior to storage, such as 3-hydroxybutan-2-one and 2-ethylhexan-1-ol, while the negative PC 1 is associated with FFA, esters, and some alcohol development. As can be seen in Figure 1, samples stored at AP/RF and 50/RT at day 7 and all samples under 75 and 100/RT are closer to the sample prior to storage (positive PC 1), while samples under AP/RF at day 14 and 50/RT at days 14 and 28 are apart from the initial one, with samples under AP/RT being the more distant ones (negative PC 1). If the same exercise is carried out with only the data set for the three major classes of identified VOC (total FFA, esters, and alcohols), a similar pattern is observed, but with a better differentiation (99.17% of total variance), with 91.95 and 7.22% of the total variance being explained by PC 1 and PC 2, respectively (Figure 2).

### 3.6 Fatty Acid Profile

The milk fatty acid profile was characterized by a greater abundance in saturated fatty acids (SFAs—63.15–63.30%), followed by monounsaturated fatty acids (MUFAs—30.28–30.92%) and polyunsaturated fatty acids (PUFAs—4.65–4.66%) (Table 3). Regarding individual fatty acids, the most abundant in the initial milk (% of total fatty acids) were palmitic acid (C16:0, 30.41 ± 0.36%), oleic acid (C18:1c, 22.33 ± 0.36%), myristic acid (C14:0, 11.26 ± 0.07%), and stearic acid (C18:0, 10.84 ± 0.06), similar to that reported for bovine raw milk in the literature,^32^ with some variations attributable to animal nutrition, seasonal feed changes, type of animal farming, or stage of lactation, among other factors.^33^ Overall, the different milk samples’ fatty acid profile did not present great changes, despite exhibiting a tendency to increase the SFA content accompanied by a decrease in both MUFAs and PUFAs over time, particularly after 60 days under 75 and 100/RT (*p* < 0.05), compared to the initial milk prior to storage. The major variations regarding 75 and 100/RT were related to the increase (*p* < 0.05) of lauric, myristic, and palmitic acids and reductions in oleic acid (±0.5%). However, when compared to storage at AP/RF after 7 days, the only significant reduction was on MUFA content after 60 days under 75/RT, presenting a significant decrease around 0.34%, which can be related to the increasing lipid oxidation values observed for that storage period (Table 1).

**Table 3:** Fatty Acid Profile of Raw Milk Prior to Storage (Initial) and Stored under the Different Storage Conditions (AP/RT, AP/RF, and 50, 75, and 100/RT) Expressed in %^a^

storage condition	Initial	AP/RT	AP/RF		50 MPa/RT		
days	0	7	7	14	7	14	28
C8:0	1.54	1.52	1.50	1.52	1.49	1.52	1.53
C9:0	0.05	0.06	0.05	0.05	0.05	0.05	0.04
C10:0	2.99	3.01	2.99	2.96	2.98	3.01	3.03
C12:0	3.67a	3.72abc	3.71ab	3.67a	3.70ab	3.72abc	3.73abc
C14:0	11.26a	11.39abc	11.36abc	11.34abc	11.32ab	11.40abcd	11.46bcd
C15:0	0.97	0.97	0.97	0.96	0.97	0.97	0.97
i-C16:0	0.02	0.03	0.02	0.02	0.03	0.03	0.03
C16:0	30.41a	30.71ab	30.76ab	30.56ab	30.74ab	30.71ab	30.75ab
ai-C17:0	0.58	0.60	0.59	0.60	0.59	0.59	0.59
C17:0	0.46	0.44	0.44	0.44	0.44	0.44	0.44
C18:0	10.84	10.89	10.94	10.79	10.92	10.87	10.89
C20:0	0.14	0.13	0.13	0.15	0.13	0.13	0.13
C21:0	0.03	0.03	0.03	0.03	0.03	0.03	0.03
C22:0	0.05	0.04	0.04	0.04	0.04	0.04	0.07
C23:0	0.03b	0.02a	0.02a	0.02a	0.02a	0.02a	0.02a
C24:0	0.03b	0.02ab	0.02ab	0.02ab	0.02ab	0.02ab	0.02a
total SFA	63.21ab	63.56abc	63.58abcd	63.20a	63.45abc	62.89abc	63.77abcd
C10:01	0.31	0.31	0.31	0.33	0.31	0.32	0.32
C12:1	0.03	0.03	0.03	0.03	0.03	0.03	0.03
C14:1t	0.21a	0.21bc	0.22bc	0.21bc	0.22bc	0.22bc	0.22bc
C14:1c	1.05a	1.07ab	1.07ab	1.06ab	1.07ab	1.07ab	1.07ab
ai-C15:1	0.48a	0.49ab	0.49ab	0.49b	0.49ab	0.49ab	0.49ab
C15:1	0.26a	0.26ab	0.26ab	0.26ab	0.26ab	0.26ab	0.26ab
C16:1t	0.05	0.06	0.05	0.05	0.05	0.05	0.05
C16:1c	1.82	1.83	1.83	1.82	1.83	1.82	1.82
C17:1	0.21	0.21	0.22	0.22	0.22	0.21	0.21
C18:1t	2.84	2.88	2.88	2.88	2.92	2.89	2.86
C18:1c	23.14b	22.99ab	22.99ab	23.08b	22.99ab	22.90ab	22.92ab
C20:1c	0.12	0.12	0.12	0.13	0.12	0.13	0.12
C24:1	0.04	0.04	0.04	0.04	0.04	0.04	0.04
total MUFA	30.66c	30.60abc	30.60bc	30.72c	30.64c	30.53abc	30.51abc
C18:2t	1.22	1.24	1.18	1.27	1.28	1.26	1.24
CLAc9,t11	0.47	0.47	0.47	0.51	0.47	0.47	0.46
CLAt10,c12	0.06	0.06	0.06	0.06	0.06	0.06	0.06
C18:2c	2.16e	2.17e	2.14cde	2.16e	2.15cde	2.12abcd	2.12abc
C18:3c6,c9,c12	0.05	0.06	0.05	0.06	0.05	0.05	0.06
C18:3c9,c12,c15	0.22	0.21	0.21	0.23	0.21	0.21	0.21
C20:2	0.05	0.05	0.05	0.03	0.05	0.04	0.04
C20:3	0.13c	0.13bc	0.13bc	0.12abc	0.12abc	0.12abc	0.12ab
C20:4	0.23d	0.21cd	0.21cd	0.20bcd	0.20bcd	0.19abc	0.16a
C20:5	0.02	0.02	0.02	0.02	0.02	0.01	0.02
C22:5	0.05	0.04	0.05	0.05	0.04	0.04	0.04
total PUFA	4.65bc	4.66bc	4.57abc	4.71c	4.66bc	4.59abc	4.53ab

storage condition	75 MPa/RT					100 MPa/RT				
days	7	14	28	39	60	7	14	28	39	60
C8:0	1.51	1.53	1.49	1.52	1.55	1.48	1.54	1.49	1.48	1.56
C9:0	0.06	0.05	0.05	0.05	0.05	0.06	0.05	0.05	0.03	0.05
C10:0	2.97	3.02	2.99	2.99	3.09	2.95	3.02	2.98	2.95	3.10
C12:0	3.67aabc	3.72abc	3.72abc	3.69a	3.80bc	3.65a	3.72abc	3.71ab	3.69a	3.82c
C14:0	11.39abc	11.44abcd	11.39abc	11.42abcd	11.58d	11.36abc	11.49bcd	11.39abc	11.42abcd	11.51cd
C15:0	0.96	0.97	0.97	0.96	0.97	0.96	0.97	0.97	0.97	0.97
i-C16:0	0.03	0.03	0.03	0.02	0.02	0.03	0.03	0.03	0.03	0.02
C16:0	30.75ab	30.79ab	30.77ab	30.83ab	30.95b	30.77ab	30.80ab	30.72ab	30.86b	30.78ab
ai-C17:0	0.59	0.60	0.59	0.59	0.59	0.59	0.59	0.59	0.59	0.59
C17:0	0.45	0.44	0.44	0.44	0.44	0.44	0.44	0.44	0.44	0.44
C18:0	10.90	10.88	10.93	10.95	10.83	10.93	10.91	10.91	10.94	10.83
C20:0	0.14	0.13	0.13	0.13	0.13	0.13	0.13	0.13	0.13	0.13
C21:0	0.03	0.03	0.03	0.03	0.03	0.03	0.03	0.03	0.03	0.03
C22:0	0.04	0.05	0.05	0.06	0.05	0.05	0.04	0.04	0.05	0.05
C23:0	0.02a	0.02a	0.02a	0.02a	0.02a	0.02a	0.02a	0.02a	0.02a	0.02a
C24:0	0.02ab	0.02ab	0.02ab	0.02ab	0.02a	0.02ab	0.02ab	0.02ab	0.02ab	0.02ab
total SFA	63.42abc	63.75bcd	63.61abcd	63.73bcd	64.12d	63.49abc	63.74cd	63.51abc	63.65abcd	63.92cd
C10:01	0.31	0.32	0.31	0.31	0.32	0.31	0.32	0.31	0.31	0.32
C12:1	0.03	0.03	0.03	0.03	0.03	0.03	0.03	0.03	0.03	0.03
C14:1t	0.21bc	0.22bc	0.22bc	0.21bc	0.22c	0.21b	0.22bc	0.22bc	0.21bc	0.22bc
C14:1c	1.06ab	1.07ab	1.07ab	1.06ab	1.08b	1.05a	1.07ab	1.07ab	1.07ab	1.08b
ai-C15:1	0.49ab	0.49ab	0.49ab	0.49ab	0.49ab	0.48ab	0.49ab	0.49ab	0.49ab	0.49b
C15:1	0.26ab	0.26ab	0.26ab	0.26ab	0.28b	0.26ab	0.26ab	0.26ab	0.26ab	0.26ab
C16:1t	0.05	0.06	0.06	0.05	0.05	0.05	0.05	0.05	0.05	0.05
C16:1c	1.82	1.84	1.82	1.81	1.81	1.82	1.81	1.82	1.82	1.81
C17:1	0.22	0.21	0.22	0.22	0.21	0.21	0.22	0.21	0.21	0.21
C18:1t	2.85	2.85	2.90	2.88	2.81	2.88	2.84	2.86	2.88	2.87
C18:1c	23.10b	22.87ab	22.94ab	22.97ab	22.69a	23.13b	22.93ab	22.88ab	22.95ab	22.68a
C20:1c	0.12	0.12	0.12	0.12	0.12	0.12	0.12	0.12	0.12	0.12
C24:1	0.05	0.04	0.04	0.04	0.04	0.03	0.04	0.04	0.04	0.04
total MUFA	30.68c	30.47abc	30.57abc	30.54abc	30.26a	30.26c	30.69abc	30.47abc	30.55abc	30.29ab
C18:2t	1.20	1.21	1.23	1.23	1.19	1.23	1.21	1.22	1.26	1.25
CLAc9,t11	0.47	0.47	0.47	0.46	0.46	0.47	0.47	0.47	0.47	0.47
CLAt10,c12	0.06	0.06	0.06	0.06	0.06	0.06	0.06	0.06	0.06	0.06
C18:2c	2.14bcde	2.11abc	2.12abcd	2.11abc	2.08a	2.16de	2.11abc	2.10ab	2.11abc	2.10ab
C18:3c6,c9,c12	0.05	0.05	0.05	0.05	0.05	0.05	0.05	0.05	0.05	0.05
C18:3c9,c12,c15	0.21	0.21	0.21	0.21	0.21	0.21	0.21	0.21	0.21	0.21
C20:2	0.05	0.04	0.04	0.04	0.05	0.05	0.04	0.04	0.04	0.05
C20:3	0.12abc	0.12abc	0.11a	0.12ab	0.12abc	0.12abc	0.12ab	0.12ab	0.11a	0.12abc
C20:4	0.19abc	0.18abc	0.20bcd	0.17ab	0.18abc	0.18abc	0.19abc	0.19abc	0.18abc	0.18abc
C20:5	0.01	0.01	0.02	0.02	0.01	0.01	0.01	0.02	0.02	0.01
C22:5	0.04	0.04	0.04	0.04	0.04	0.04	0.04	0.04	0.04	0.04
total PUFA	4.56abc	4.52ab	4.53ab	4.51ab	4.44a	4.59abc	4.51ab	4.51ab	4.55abc	4.54ab

a Different letters (a–d) indicate significant differences (*p* < 0.05) between the different storage conditions. Standard deviation is at least below 10% of the mean value and thus is not displayed in the table.

### 3.7 Secondary Lipid Oxidation Byproducts

Lipid oxidation is responsible for the production of numerous undesirable compounds that impact negatively the sensory and nutritional qualities of dairy products, which can be enhanced by the presence of oxygen, light, endogenous, and exogenous metals and enzymes. The MDA content was monitored as an indicator of secondary lipid oxidation development in all milk samples, being the results presented in Table 1. The initial value (0.83 ± 0.08 μg MDA/mL) observed is similar to the ones reported by Johnson et al.,^34^ and the only storage condition that presented a significant change (increase) was AP/RT, reaching values of 1.18 ± 0.07 μg MDA/mL (*p* < 0.05) after 7 days. All the other storage conditions showed no significant (*p* > 0.05) variations in lipid oxidation values, despite showing a tendency to increase in HS samples over time. This resulted in a good maintenance of MDA values for all HS samples up to 60 days of storage (particularly at 75 and 100 MPa), compared to the values of the initial milk, because after 7 and 14 days of storage, these samples showed even lower MDA concentration. Noteworthy is the fact that after 60 days at RT, the samples stored under 75 and 100 MPa presented TBARS values (1.03 ± 0.11 and 0.84 ± 0.05 μg MDA/mL, respectively) below 1.3 μg MDA/mL, which was associated with the perceptible sensory changes in milk reported in Johnson et al.’s^34^ work.

### 3.8 Protein Profile

Total protein was quantified prior to and after storage at the different conditions (Table 4), with an initial value of 3.42 ± 0.13 g/100 mL, which is in accordance with the literature for bovine milk;^15^ no variations (*p* > 0.05) were observed between all the different storage conditions, even after 60 days at 75 and 100/RT.

**Table 4:** Total Protein (g/100 mL), SP (mg/100 mL), and FAAs (nmol/mL) of Raw Milk Prior to Storage (Initial) and Stored under the Different Conditions (AP/RT, AP/RF, and 50, 75, and 100/RT)^a^

condition	Initial	AP/RT	AP/RF		50 MPa/RT			75 MPa/RT					100 MPa/RT				
days	0	7	7	14	7	14	28	7	14	28	39	60	7	14	28	39	60
total protein (g/100 mL)	3.42	3.57	3.52	3.56	NP	3.36	3.37	NP	3.52	3.43	NP	3.36	NP	3.37	3.39	NP	3.38
SP (mg/100 mL)	1.89bcd	1.06a	1.20a	1.24a	1.07a	1.48ab	1.52ab	1.78bc	2.24cde	2.38de	3.19fg	7.92h	1.90bcd	2.74ef	3.04f	3.69g	11.10i

condition	Initial	AP/RT	AP/RF		50 MPa/RT			75 MPa/RT					100 MPa/RT				
days	0	7	7	14	7	14	28	7	14	28	39	60	7	14	28	39	60
FAA (nmol/mL)																	
alanine	146.0a	321.8cd	295.9bcd	355.7d	NP	546.4e	675.6f	NP	271.7bc	303.6bcd	NP	546.0e	NP	251.5b	316.2cd	NP	651.7f
glycine	103.7b	74.4b	20.0a	28.0a	NP	35.6a	38.4a	NP	144.3c	143.1c	NP	268.6e	NP	150.4c	173.2c	NP	372.3e
valine*	64.1a	70.2a	30.8a	43.9a	NP	279.6b	339.2bcd	NP	352.8bcd	415.0cd	NP	990.9e	NP	290.9cd	424.7d	NP	1115.3e
leucine*	38.0a	86.5ab	114.7ab	120.9ab	NP	188.8bc	425.0d	NP	265.1c	485.4de	NP	1325.3f	NP	293.3c	580.2e	NP	1502.0g
isoleucine*	24.7ab	9.6a	12.6a	10.1a	NP	103.3bc	126.6cd	NP	129.7cd	169.7cd	NP	582.3e	NP	129.0cd	202.7d	NP	666.5i
threonine	16.4a	12.6a	12.0a	13.3a	NP	41.0b	45.1b	NP	50.0b	73.6c	NP	250.3e	NP	87.9c	137.3d	NP	393.5f
serine	16.7a	94.4def	32.3ab	34.3ab	NP	62.4bc	71.0cde	NP	63.0bcd	98.7ef	NP	429.7h	NP	112.1f	166.5g	NP	547.7i
proline	63.0ab	152.9c	61.8a	77.5ab	NP	142.5c	124.8bc	NP	119.2bc	138.5c	NP	423.2e	NP	134.1c	232.5d	NP	778.7f
asparagine	8.6ab	12.9b	10.0ab	6.2a	NP	10.2ab	8.7ab	NP	8.1ab	7.8ab	NP	21.20c	NP	6.8ab	9.8a	NP	24.2c
aspartic acid	62.0a	155.2a	95.6a	133.0a	NP	86.5a	114.4a	NP	69.1a	92.6a	NP	324.9b	NP	71.1a	136.2a	NP	498.8c
methionine*	6.2a	3.3a	1.4a	3.0a	NP	5.5a	6.1a	NP	37.0b	57.8c	NP	203.6e	NP	52.4c	95.2d	NP	295.5f
hydroxyproline	12.1ab	13.7ab	10.5ab	13.2ab	NP	10.2ab	6.0a	NP	15.0b	13.3ab	NP	16.9bc	NP	12.1ab	13.4ab	NP	24.3c
glutamic acid	585.8a	679.2ab	767.6ab	874.4ab	NP	609.3a	645.8a	NP	673.7ab	903.4ab	NP	2058.7c	NP	801.5ab	1031.6b	NP	1777.1c
phenylalanine*	19.5a	10.7a	1.4a	0.6a	NP	28.0a	34.6a	NP	87.6b	149.1c	NP	426.6d	NP	101.0b	176.7c	NP	503.8e
ornithine	18.0abcd	41.0e	15.4abc	11.6a	NP	18.5abcd	33.1de	NP	10.4a	12.7ab	NP	18.5abcd	NP	15.4abc	28.0bcde	NP	28.4cde
lysine*	22.6ab	13.0a	34.6ab	30.7ab	NP	36.9ab	50.6bc	NP	25.9ab	46.9bc	NP	81.0d	NP	30.0ab	32.2ab	NP	68.8cd
histidine*	72.0a	22.4a	14.8a	16.9a	NP	73.7a	82.6a	NP	310.7b	395.0b	NP	891.9d	NP	423.2b	575.6c	NP	1119.2e
tyrosine*	2.6a	ND	2.8a	3.5a	NP	1.3a	1.1a	NP	5.8a	22.0a	NP	93.4c	NP	12.9a	54.6b	NP	160.7d
tryptophan*	18.4a	19.3a	3.5a	3.6a	NP	30.5a	54.5ab	NP	96.0b	209.1c	NP	538.1d	NP	107.3b	250.2c	NP	665.8e
cystine	11.7a	19.8a	9.9a	10.7a	NP	8.8a	14.4a	NP	14.1a	56.5b	NP	198.9c	NP	20.7a	79.3b	NP	208.6c
total FAA (μmol/mL)	1.3a	1.8ab	1.6ab	1.8ab	NP	2.3bc	3.0cd	NP	2.8c	3.9de	NP	10.1f	NP	3.1cd	4.7e	NP	11.4g

a Different letters (a–i) indicate significant differences (*p* < 0.05) between the different storage conditions for each parameter. In general, the standard deviation is at least below 10% of the mean value and thus is not displayed in the table. NP—parameters not performed under these conditions. ND—not detected, *—essential amino acids.

Regarding SP, an overall increase (*p* < 0.05) was observed, especially for longer storage periods (Table 4). The SP content for initial milk was 1.89 ± 0.10 mg/100 mL, decreasing after 7 days (*p* < 0.05) at AP/RF, 50/RT, and AP/RT to a minimum of 1.06 ± 0.10 mg/100 mL, possibly related to nitrogen uptake for microbial metabolism. Storage under 75 and 100/RT maintained SP concentration after 7 days (*p* > 0.05), which tended to increase as the storage period increased, with a significant variation being observed from day 14 to 39 (*p* < 0.05) of storage, under 75 and 100/RT, respectively. Especially, from day 39 until the end of the study, the SP concentration had a pronounce increase of almost 4- to 6-fold under 75 and 100/RT, respectively, which can be attributed to the enzymatic activity of proteases such as plasmin and microbial proteases.^22^ The SP increment in milk can occur in prolonged storage at RT, with Olfa et al.^22^ reporting an increase of 140% from day 0 to 60 days at 30 °C in the SP content of UHT milk, possibly as a result of thermoresistant microbial proteases (mainly from*Pseudomonas* spp.) since plasmin tends to lose most of its activity at UHT conditions.^35^ The HPP of raw milk^36^ seems to affect only the plasmin activity for pressures higher than 250 MPa (10–30 min), which decreased throughout the storage at AP/RF and at 37 °C but in the end resulted in a significant increase in SP content, even in treatments with reduced plasmin activity, thus pointing to the involvement of other proteases in this process.

FAAs showed a similar behavior during the study as to what was observed to SP, under the different storage conditions (Table 4), with a linear correlation being observed between SP and FAAs (SP = FAA × 0.001 – 0.302, *r*^2^ = 0.968). Initially, raw milk had a total FAA of 1.3 ± 0.1 μmol/mL, being detected 20 aa, with all the essential ones being present and characterized mainly by a high abundance of glutamic acid, alanine, glycine, histidine, and proline, which is in accordance with the literature.^37^ Storage under AP/RT and AP/RF resulted in similar total FAA values (*p* > 0.05), compared to the initial values, despite the tendency to increase at the end of storage for these two conditions. On the other hand, HS presented an overall considerable increase throughout the storage on the majority of FAAs, increasing over time and were more pronounced with the increase of pressure (*p* < 0.05). Milk stored under 75 and 100/RT had a similar FAAs profile, but compared to the initial FAAs, a greater variance (*p* < 0.05) was observed especially for tyrosine > methionine > leucine > tryptophan > serine > isoleucine > phenylalanine > threonine > cystine > valine > and histidine, with these FAAs being associated with plasmin and microbial protease enzymatic activity on caseins.^22^ Since in this study, raw milk without any kind of processing was employed, a variety of active microbial proteases can be present initially, alongside with endogenous plasmin, which can promote casein proteolysis into small peptides or amino acids.^35^

The information regarding the effect of HPP in milk plasmin and other proteases usually employed higher pressures (200–400 MPa) than the ones used in this study, and for shorter periods of time, and thus, it is difficult to make a straightforward comparison of the results obtained in these different conditions.^36^ However, when Atlantic salmon was stored under HS conditions (50–75 MPa at 10–25 °C), several proteases (cathepsin B, D, and calpains) have been shown to maintain partial activity even after periods up to 50 days;^20^ these protease activities are more affected by the storage temperature than by the HS pressure level during storage, with storage at lower temperature (10 °C) causing changes, to a lesser extent in the myofibrillar fragmentation index.^20^

Overall, the quality and nutritional parameters of raw milk evaluated in this study point to a better preservation by HS, compared to conventional RF, particularly for pressures of 75 and 100 MPa. For instance, the only parameter, from those studied, found to be considerably affected by HS was the FAA content, indicating a higher proteolytic activity after 60 days under HS. Thus, further research regarding the HS effect on the proteolytic agents of raw milk should be investigated in order to fully understand it and its impacts on the sensorial properties of milk and the possible technofunctional properties in the latter on produced dairy products. All the other parameters monitored indicate a better preservation under HS (75–100/RT) of raw milk, resulting in an overall similar profile to raw milk prior to storage by instrumental analysis, retaining the characteristic fatty acids and VOC profiles, and corroborated by a PCA for the latter, clearly resulting in a better preservation methodology compared to RF for longer storage periods (similar observations were found for milk microbial quality in another work).^6^

In conclusion, HS at 75 and 100 MPa at RT is a clear promising food preservation methodology for storage of raw milk, leading possibly to considerable shelf life extension with an overall closer quality, similar to raw and refrigerated milk (but in this case for a much shorter storage period) and should be further studied, given the high importance of milk in the human diet.
